# Microbial Response to Phytostabilization in Mining Impacted Soils Using Maize in Conjunction with Biochar and Compost

**DOI:** 10.3390/microorganisms9122545

**Published:** 2021-12-09

**Authors:** Thomas F. Ducey, Gilbert C. Sigua, Jeffrey M. Novak, James A. Ippolito, Kurt A. Spokas, Mark G. Johnson

**Affiliations:** 1 Coastal Plains Soil, Water, and Plant Research Center, ARS-USDA, Florence, SC 29501, USA; gilbert.sigua@usda.gov (G.C.S.); jeff.novak@usda.gov (J.M.N.); 2 Department of Soil and Crop Sciences, Colorado State University, Fort Collins, CO 80523, USA; jim.ippolito@colostate.edu; 3 National Forage Seed Production Research Center, ARS-USDA, St. Paul, MN 55105, USA; kurt.spokas@usda.gov; 4 Center for Public Health and Environmental Assessment, Pacific Ecological Systems Division, United States Environmental Protection Agency, Corvallis, OR 97333, USA; johnson.markg@epa.gov

**Keywords:** poultry litter biochar, beef cattle manure biochar, microbial structure and function, heavy metals, cadmium, zinc

## Abstract

Even after remediation, mining impacted soils can leave behind a landscape inhospitable to plant growth and containing residual heavy metals. While phytostabilization can be used to restore such sites by limiting heavy metal spread, it is reliant on soil capable of supporting plant growth. Manure-based biochars, coupled with compost, have demonstrated the ability to improve soil growth conditions in mine impacted soils, however there is a paucity of information regarding their influence on resident microbial populations. The objective of this study was to elucidate the impact of these soil amendments on microbial community structure and function in mine impacted soils placed under phytostabilization management with maize. To this aim, a combination of phospholipid fatty acid (PLFA) and enzymatic analyses were performed. Results indicate that microbial biomass is significantly increased upon addition of biochar and compost, with maximal microbial biomass achieved with 5% poultry litter biochar and compost (62.82 nmol g^−1^ dry soil). Microbial community structure was impacted by biochar type, rate of application, and compost addition, and influenced by pH (*r*^2^ = 0.778), EC (*r*^2^ = 0.467), and Mg soil concentrations (*r*^2^ = 0.453). In three of the four enzymes analyzed, poultry litter biochar treatments were observed with increased activity rates that were often significantly greater than the unamended control. Overall, enzyme activities rates were influenced by biochar type and rate, and addition of compost. These results suggest that using a combination of biochar and compost can be utilized as a management tool to support phytostabilization strategies in mining impacted soils.

## 1. Introduction

Mining activity serves as one of the primary sources of heavy metal accumulation in soils, the results of which pose serious, long-term concerns for human and environmental health. To mitigate these deleterious impacts, a variety of strategies to either remove or sequester heavy metals have been adopted. Phytoremediation is a series of plant-driven processes with the goal of reducing heavy metal concentrations either by extracting them from the soil (e.g., phytoextraction), or by reducing their bioavailability (e.g., phytostabilization) [1]. When coupled with other tools, such as biochar, designed to reduce heavy metal bioavailability, the combinative impact of these dual strategies can result in improved remediation efforts [2,3]. This synergy is often attributable to microbial communities aptly suited to the remediation environment. Microbial-related modes of action include: (i) direct sequestration of heavy metals [4]; and/or (ii) enhanced plant-mediated heavy metal reductions spurred by plant growth promotion or alterations to the rhizospheric environment [5,6]. One caveat to this scenario is that unless these microbial communities are directly introduced during the remediation process [7], they must either be native to the soil being remediated, have colonized the biochar prior to use as a soil amendment, or be found in other organic treatments being utilized. Unfortunately, soils contaminated with high concentrations of heavy metals are often compromised, and exhibit reduced microbial biomass, lower microbial diversity, and decreased microbial enzymatic activities [8,9,10]. These results indicate that relying on native soil microbial populations to assist biologically driven remediation processes may be proven problematic [11], though potentially site-specific [12].

Studies have indicated that soil amendments, such as biochar and compost, have the potential to recondition mining impacted soils, resulting in increased microbial survivability [13]. A phytoextraction study by Fornes et al. [14] involving several *Brassica* species demonstrated that compost amendment could significantly increase microbial activity in heavy metal contaminated calcareous soils. Inversely, this same study demonstrated that in acidic soils, an identical compost treatment did not improve microbial activity, potentially due to Al solubility and toxicity [14], highlighting the importance of properly selecting treatment materials (e.g., biochar, compost, lime, etc.) to improve soil functionality in support of remediation efforts. In regard to biochar specifically, Novak et al. [15] painstakingly detailed the concept of “designer biochar” to specifically target problematic physicochemical soil characteristics while avoiding unintended and wholly unfavorable impacts to soil quality. Ducey et al. [16] further expanded this concept to cover biological soil characteristics, by demonstrating the complex relationship between microbial community structure as influenced by biochar feedstock and soil type.

The soils used in this study originate from the Oronogo–Duenweg Mining Belt, located in Jasper County, MO, USA. This location was previously home to lead (Pb) and zinc (Zn) mining operations. After Pb and Zn extraction, non-ore waste rock and tailings were deposited into what is conventionally referred to as “chat piles”. Leaching of heavy metals from these chat piles has led to contamination of the underlying soil with high levels of Pb, Zn, and cadmium (Cd). The severity of this contamination led to placement of the Oronogo-Duenweg Mining Belt onto the National Priorities List in 1990, thereby mandating EPA-directed remediation efforts. Remediation consists of removing chat piles, and Pb-contaminated topsoil until Pb levels reflect background concentrations [17]. Unfortunately, at many of these sites, considerable concentrations of Cd and Zn remain. Additionally, in the course of remediation, Pb-contamination proved so pervasive that the soil O, A, and B horizons were removed. The resulting remediated landscape proved undesirable for private landowners on whose land much of this heavy metal contaminated soil was found, frustrating remediation efforts. Therefore, efforts were undertaken to include various restoration strategies, to restore soil quality and revegetate the landscape to complement remediation.

Novak et al. [18] utilized the designer biochar approach on these mining impacted soils, demonstrating that three different biochars, coupled with compost, could significantly decrease bioavailable Cd and Zn concentrations, and allowed for growth of switchgrass. Additionally, Ducey et al. [19] reported that two manure-derived biochars supported growth of two grasses, *Panicum virgatum* (switchgrass) and *Bouteloua dactyloides* (buffalograss), and that grass type significantly influenced microbial biomass, activity, and community structure. Sigua et al. [20] further expounded on this biochar research to support phytostabilization efforts with maize (*Zea mays* L.). Given Ducey et al.’s [19] previously documented influence of plant type on microbial structure and function in this mining impacted soil, it is critical to understand the dynamics between microbes, biochar, compost, and maize; to date, there is a paucity of relevant data. Despite this knowledge gap, given prior results with other plant species, we hypothesize that microbial populations will respond favorably to biochar and compost amendment under maize phytostabilization in these mine impacted soils.

The primary goal for these studies was to generate data sufficient to expand our knowledge base, and to offer sound management guidance on remediation and restoration efforts within the Oronogo–Duenweg Mining Belt. The aim of the current project was to understand the impact of two manure-based biochars on microbiological properties of a mining impacted soil planted with maize for phytostabilization purposes. Based on prior studies, the biochars selected for this study were derived from weathered beef cattle manure and poultry litter feedstocks, applied at two rates (2.5% and 5%), coupled, or not, with a manure-based compost. Treatments were examined for impacts on microbial biomass, structure, and function.

## 2. Materials and Methods

### 2.1. Site Description, Soil and Biochar Preparation

For the collection of mine impacted soils, a site was selected from within the Oronogo-Duenweg mining area adjacent to Webb City in Jasper County, MO, USA. Due to remediation efforts, the site’s O, A, and B soil horizons have been removed, resulting in exposure of the C horizon at the surface. The C horizon is composed of an amalgamation of gravelly silt loam, cobbly clay, and 2 to 15 cm sized rock fragments in the exposed subsoil. This subsoil was collected via excavation with a tractor mounted backhoe, and placed into plastic-lined drums [≈190 L] prior to shipment to the Agricultural Research Service’s (United States Department of Agriculture) Coastal Plains Soil, Water, and Plant Research Center, located in Florence, SC. Due to the presence of large rock and coarse fragments, subsequent to air-drying, the soil material was sieved using a 12.7 mm diameter screen to collect material more appropriate for use in the potted greenhouse study. This sieving revealed that roughly a third of the collected soil material consisted of fragments greater than 12.7 mm in size [18,20]. This sieved, subsoil material will henceforth be referred to as mining impacted soils.

Two biochars were utilized in this study. The first biochar was derived from a beef cattle manure (BC) feedstock, collected from a local feedlot in Webb City, MO, USA. This manure was mixed 1:1 with locally sourced green waste, weathered for two years, sieved (6 mm) and pyrolyzed at 500 °C with a residence time of approximately four hours [15]. The second biochar was commercially sourced, utilizing poultry litter (PL) as a feedstock, and gasified under a proprietary process. For a complete analysis of feedstock and biochar properties, please refer to Novak et al. [18] and Sigua et al. [20].

### 2.2. Experimental Design

This experiment was a smaller component of a larger study designed as a pot study performed under greenhouse conditions, and is described in full by Sigua et al. [20]. Treatments consisted of two biochar additions: beef cattle manure (BC); and poultry litter (PL). Biochars were added as soil treatments at 2.5% or 5.0% *w*/*w* rates and served as either the lone soil amendment (0% compost) or were coupled with compost applied at a 5% *w*/*w* rate. Control treatments *sans* biochar, with or without 5% compost (i.e., unpyrolyzed BC feedstock) addition were also included in the study. Amendment selection and application rates were based on a previous study by Ducey et al. [19] that demonstrated the efficacy of these treatments to grow native grasses in the same mining impacted soils. Each treatment combination was performed in triplicate, in 15 cm (top diameter) × 17 cm (depth) pots. Each pot received a total of 1.5 kg of mine soil treated at their respective rates, and compacted to a bulk density of 1.5 g/cm^3^ [21]. Pots were randomly arranged on greenhouse tables. A total of eight maize seeds were planted in each pot, fertilized with NH_4_NO_3_ (equivalent to 25 kg N ha^−1^) on day 16 to prevent N deficiency, and watered to field capacity several times a week. Greenhouse conditions during the experiment were as follows: mean air temperature 21.8 ± 3.1 °C; and mean relative humidity of 53 ± 12.2%. The experiment was halted at the point that the plants became root bound, which occurred at day 35. Pots were destructively sampled upon conclusion of the study. Fresh soil in 3 g and 15 g allotments were set aside for soil enzyme activity assays and PLFA analysis, respectively. The remaining soil was air dried and passed through a 2 mm sieve prior to determination of pH and water extractable nutrient concentrations.

### 2.3. Soil Analysis 

Soil pH and EC were measured using standard protocols as outlined [22], with a slight modification for pH determination, in relation to *w*:*w* ratios, as mentioned in [15]. Water-extractable soil nutrient and metal concentrations were determined in triplicate using inductively coupled plasma-optical emission spectroscopy (ICP-OES) similar to the procedure of Bai et al. [23]. Briefly, 30 g of soil was mixed with 60 mL of deionized water for 30 min, allowed to settle, and filtered through a 0.45 μm syringe filter prior to analysis.

### 2.4. Phospholipid Fatty Acid (PLFA) Analysis

At the termination of the study, pots were destructively sampled and a total of 15 g of fresh soil from each pot was lyophilized according to Veum et al. [24], and shipped overnight on dry ice to MIDI Inc. (Newark, DE, USA) for high-throughput PLFA analysis according to the methodology established by Buyer and Sasser [25]. Briefly, lyophilized soil was extracted with Bligh–Dyer extractant and the resultant liquid phase was separated with chloroform and deionized water. The upper, aqueous phase was aspirated and discarded, and the bottom lipid-containing phase was collected, evaporated at 30 °C, and stored overnight at −20 °C. Lipid classes were separated using solid phase extraction (SPE) in a 96-well format using silica. Samples were dissolved in chloroform, passed through the SPE plate, and phospholipids were eluted with 5:5:1 methanol:chloroform:H_2_O. The solution was evaporated at 70 °C for 30 min followed by 37 °C until completely dry. Transesterification reagent was added to the samples, incubated for 15 min at 37 °C, followed by addition of 0.075 M acetic acid and then twice extracted with chloroform. Chloroform was removed by drying, and lipids were dissolved in hexane for analysis on a gas chromatograph with a flame ionization detector. FAMEs were separated on an Agilent Ultra 2 column, 25 m long × 0.2 mm internal diameter × 0.33 μm film thickness using hydrogen gas as the carrier with a flow rate of 1.22 mL min^−1^. FAMEs were identified using the MIDI PFALDI calibration mix and naming table [25]. For both microbial biomass and community analysis, PLFAs with retention rates between C14:0 and C22:0 were included in the data analysis [16]. For community analysis, PLFAs were normalized as a ratio to C16:0 [26], to account for differences in extraction efficiencies between biochar amendment rates [27]. After normalization, PLFAs with ratios below 0.02 in greater than 90% of the samples, were omitted from the data set. This resulted in removal of 8 PLFAs (28 remaining, excluding C16:0) from analysis.

### 2.5. Soil Enzyme Assays

Enzymatic activity of three extracellular enzymes—β-glucosidase (BG), N-acetyl-β-d-glucosaminidase (NAG), and acid phosphomonoesterase (AcP)—and one intracellular enzyme—esterase (EST)—were measured by fluorimetric microtiter plate assay according to Deng et al. [28,29]. Briefly, upon termination of the study, two 1 g subsamples of soil were removed from each sample and mixed in 150 mL deionized water using a magnetic stir bar at 600 rpm. After mixing for 30 min, 100 μL aliquots of soil slurry were added to black, flat-bottomed wells of a 96-well plate (ThermoFisher, Waltham, MA, USA) and incubated at 37 °C for 1 h in a water-jacketed incubator. To each well the following was also added: 50 μL Modified Universal Buffer (pH 5.5 for NAG, pH 6.0 for all others) and 50 μL of the respective methylumbelliferyl-linked enzyme substrate (MilliporeSigma, Burlington, MA, USA) as follows: BG, 4-methylumbelliferyl-β-d-glucoside; NAG, 4-methylumbelliferyl-N-acetyl-β-glucosaminide; AcP, 4-methylumbelliferyl-phosphate; EST, 4-methylumbelliferyl -butyrate. After incubation, enzymatic reactions were halted by addition of 50 μL of 0.1 M THAM (pH 12). Fluorescence was measured at 360 nm excitation and 460 nm emission, on a Biotek FLx800 plate reader connected to a laptop equipped with Gen5 data acquisition software (Biotek, Winooski, VT, USA). The collected raw data were exported to a Microsoft Excel spreadsheet (Microsoft Corporation, Redmond, WA, USA) for processing. Standard curves for each soil suspension were prepared using methylumbelliferone standards (MilliporeSigma, Burlington, MA, USA) in concentrations ranging from 0 to 50 μM. Determination of autohydrolysis was determined by incubating substrate in deionized water and was used to correct final enzyme activity rates. Any resultant negative assay values were removed prior to statistical analysis. Each of the two subsamples were assayed in triplicate, for a total of six data points per sample, for each enzyme.

### 2.6. Statistics

Analysis of variance and correlation analyses were performed using Minitab (ver 20.4; Minitab Incorporated, State College, PA, USA). All data were first tested for normal distribution using the Ryan–Joiner test, while error variances were assessed using the modified Levene’s test for equality. Biochar (type and rate) and compost (with or without) were the fixed variables while pH, EC, soil Cd and Zn concentrations, microbial biomass, and BG, NAG, AcP, and EST enzyme activity rates were the dependent variables in a two-way ANOVA. Pairwise comparisons were calculated using Fisher’s Least Square Difference Method (LSD). Biological data (microbial biomass, and all four enzyme activity rates) and EC were non-normal and log transformed prior to ANOVA. Soil pH, and Cd and Zn concentrations did not violate assumptions of normality or homogeneity of variance and were not transformed. Differences between means were considered significant at *p* < 0.05. All usage of the word “significant” throughout the text implies *p* < 0.05. Non-metric multidimensional scaling (NMS) of microbial community population data was performed in PC-Ord v.6 (MJM Software Design, Gleneden Beach, OR, USA), with a secondary matrix containing soil pH, soil electrical conductivity, soil Cd and Zn concentrations, and other water extractable elements, data that is provided in Supplementary Table S1.

## 3. Results and Discussion

### 3.1. Post-Treatment Soil Analysis

**Figure 1:**
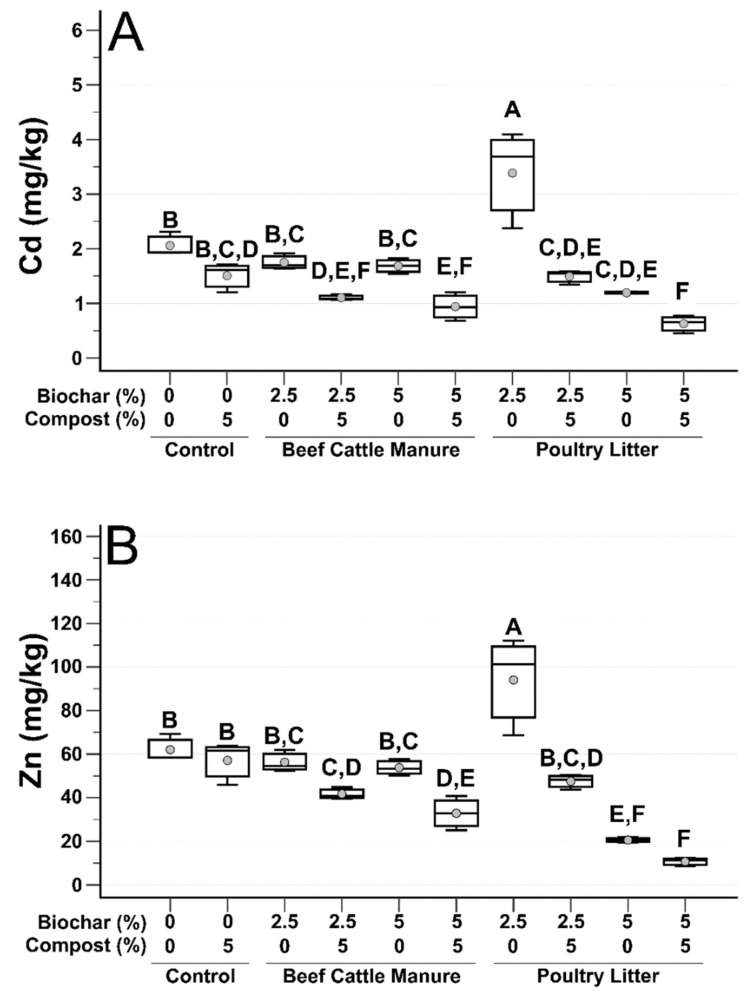
Box and whisker plots of soil Cd (**A**) and Zn (**B**) post treatment. Gray circles are mean values, top and bottom of boxes are 75th and 25th percentile, respectively, median is center line, and whiskers are range of the data. Treatments that do not share a letter are significantly different (*p* < 0.05).

Figure 1 shows water-extractable Cd and Zn soil concentrations (mg/kg), post treatment. For Cd soil concentrations (Figure 1A), five of the eight biochar treatments were significantly lower than the unamended (0% biochar/0% compost) control. Of those five treatments, four included a compost amendment. Amongst the no biochar treatments, compost alone lowered Cd soil concentrations, however it did not do so significantly compared to the unamended control. Treatments that included PL biochar at 5% (*w*/*w*) resulted in significantly decreased Cd concentrations compared to the unamended control. PL biochar at 5% amendment rate, coupled with compost resulted in the greatest decrease in soil Cd concentrations.

Similar to Cd soil concentrations, compost additions to biochar significantly decreased Zn soil concentrations (Figure 1B) in three of four treatments, the exception being PL 2.5% with compost. Zn soil concentrations were the lowest when PL biochar was utilized at a 5% amendment rate. As with Cd, PL biochar at 5% amendment rate, coupled with compost, resulted in the greatest decrease in soil Zn. It should also be pointed out that for both Cd and Zn, mine impacted soils treated with 2.5% PL without compost resulted in a significant spike in heavy metal concentrations, even over the unamended control soil. PL biochar has been demonstrated to have liming capabilities in the past [15], however at a biochar amendment rate of 2.5%, soil pH remained acidic (Table 1). Additionally, soil EC significantly increased when PL biochar was added to the mine impacted soils (Table 1), and salinity has been demonstrated to contribute to increased heavy metal solubility [30]. In this study, PL biochar added a considerable amount of sodium (Na) to the mining impacted soils (see Supplementary Table S1). At the 2.5% biochar rate, without significantly raising the pH, heavy metal solubility could still be countered by the addition of organic matter supplied by compost [31,32]. This was observed in the 2.5% PL biochar/5% compost treatment (Figure 1), indicating that amendment rate has an impactful role in reducing water-soluble Cd and Zn soil concentrations.

Overall, these results are similar to the results of Novak et al. [18] and Ducey et al. [19] in these same mining impacted soils. While these studies reported different plant species (switchgrass and buffalograss, respectively), they demonstrated the ability of manure-derived biochars to reduce Cd and Zn soil concentrations with increasing biochar and compost application rates, while concomitantly improving soil conditions to allow for plant growth. For this study, plant growth is reported extensively in Sigua et al. [20] that demonstrated that both biochar type and compost amendment rate significantly increased shoot biomass over the control soil.

### 3.2. Microbial Biomass

**Figure 2:**
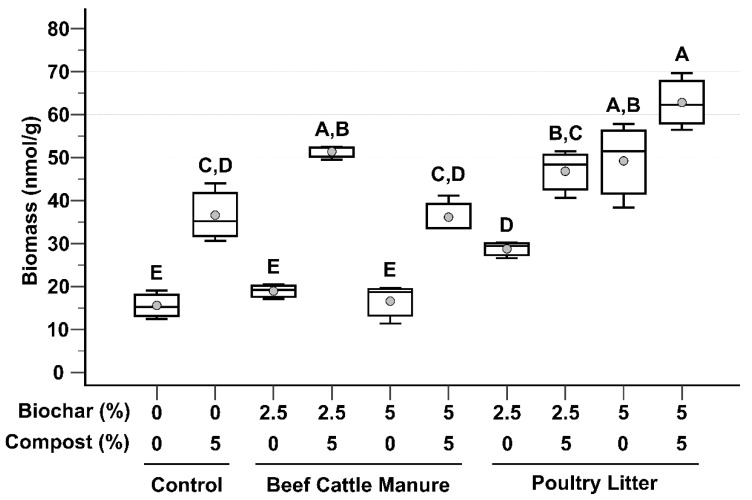
Box and whisker plots of total microbial biomass as measured by PLFA. Gray circles are mean values, top and bottom of boxes are 75th and 25th percentile, respectively, median is center line, and whiskers are range of the data. Treatments that do not share a letter are significantly different (*p* < 0.05). Data are presented as untransformed, with statistical analysis based on log-transformed values.

Microbial biomass, as determined by PLFA, is shown in Figure 2. When comparing treatments, BC biochar alone, regardless of amendment rate, did not lead to increased microbial biomass values when compared to the unamended control soil. On the other hand, PL biochar alone treatments did lead to significant increases. Compost had a marked impact on microbial biomass, leading to significant increases in microbial biomass for all treatments except PL 5%. However, compost addition to PL 5% did trend upward when compared to PL 5% without compost (Figure 2).

Comparison between the two biochar amendments revealed disparate responses in microbial biomass. With the exception of the 2.5% biochar/5% compost treatments which resulted in similar levels of microbial biomass, all PL biochar treatments led to significantly greater microbial biomass values (Figure 2) compared to their BC biochar counterparts. The PL 5% with compost treatment resulted in the highest microbial biomass (mean: 62.82 nmol g^−1^ dry soil), with the unamended control having the lowest (mean: 15.6 nmol g^−1^ dry soil). These results are identical to those reported by Ducey et al. [19] for buffalograss. It is possible that use of fresh, as opposed to weathered, beef cattle manure would have more closely resembled the poultry litter biochar treatments, at least regarding microbial biomass. Biochar chemical composition, as reported by Sigua et al. [20], reveals that the BC biochar has lower C, N, and P compared to the PL biochar, thereby potentially limiting the amount of nutrients available for microbial growth.

Numerous studies have demonstrated positive relationships between biochar amendment and microbial biomass [33,34,35]. Negative impacts can often be explained by adverse adjustments to soil physicochemical characteristics, such as pH [36]. When great care is taken in designing a biochar to specifically ameliorate known soil physicochemical and biological defects, results tend to be overwhelmingly positive, as evidenced by our current line of research on mining impacted soils, manure-based biochars, and compost. The beneficial impacts on microbial biomass by biochar have also been demonstrated by others. Kumar et al. [37] and Masto et al. [38] both reported that use of biochar made from invasive weeds, when added to agricultural soils, resulted in significant microbial biomass increases that corresponded to biochar amendment rate. These results led Ghosh and Maiti [39] to hypothesize that such biochars could be used to remediate coal mine spoils.

In order for phytoremediation management to work effectively, the soil must provide a matrix capable of sustaining the plants of choice [40]. There are many modes of action by which these processes can be microbially-enhanced [3]. In many of these cases, given the strong relationship between microbial biomass and soil fertility [41], it is reasonable to hypothesize that treatments, such as biochar and compost, that increase microbial biomass will have a concomitant, positive impact on phytoremediation processes.

### 3.3. Soil Microbial Community Structure

**Figure 3:**
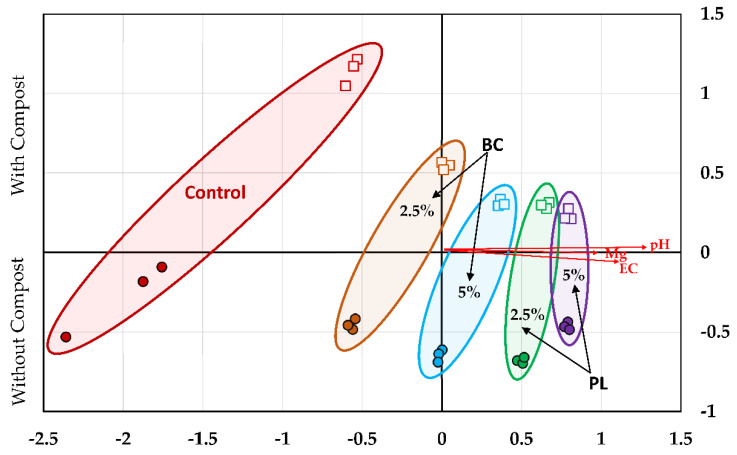
Nonmetric multidimensional scaling plot showing mining impacted soil microbial community structure. Symbols represent compost treatments, which are also labeled on the left Y axis. Circles are treatments without compost, while open squares are treatments with compost. Labels differentiate biochar treatments and are encompassed by ellipses. BC = beef cattle manure biochar; PL = poultry litter biochar; control = non-biochar amended soils. Joint plot vectors (red line) were selected for display based on a combined *R*^2^ cutoff of 0.45 or greater.

Microbial community structure was assessed by nonmetric multidimensional scaling analysis (Figure 3). Microbial communities with similar structures are depicted as points that cluster closer together. Changes in biochar treatment resulted in movement along the X axis. PL biochar had the largest shift away from the control soils (located on the left side of the graph), while BC was situated between PL and the control. Neither biochar treatment had any overlap with the non-biochar treated controls. Compost treatment is clearly demarcated along the Y axis. For a majority of the treatments, clustering of samples receiving the same treatment are tight, indicating low variation in the PLFA composition between those samples.

Shifts in microbial community structure were incremental; increases in biochar amendment rate resulted in further shifts away from the unamended controls along the X axis (Figure 3). Several strong relationships (*r*^2^ > 0.45) amongst microbial communities and physicochemical properties were observed. These relationships were all significantly correlated along the X axis with pH (*r*^2^ = 0.778), EC (*r*^2^ = 0.467), and Mg (*r*^2^ = 0.453). No measured soil physicochemical parameters were significant along the Y axis. The relationship between pH and microbial community structure is not surprising, as pH is one of the main drivers of soil microbial diversity [42]. This result has also been observed in the previously mentioned, related study by Ducey et al. [19], using switchgrass and buffalograss grown in the same mining impacted soil. In addition to driving changes in microbial community structure directly, increases to soil pH can result in decreases to heavy metal solubility [43]. In turn, these decreased concentrations could potentially lead to lowered heavy metal-related stress on resident microbial populations [44].

Electrical conductivity has also been linked to changes in microbial community structure in other studies [45], which is potentially explained by the increased concentrations of Na found in PL biochar (Supplementary Table S1; NMS correlation *r*^2^ = 0.437). Additionally, magnesium has similarly been reported to be a driver of changes to microbial community structure in cultivated systems, primarily due to linkage with nutrient availability for plant growth [46]. In a study by Hou et al. [47], they hypothesized that Mg concentrations correlated with bacterial groups capable of metabolizing recalcitrant C substrates, a plausible scenario in biochar-amended soils.

### 3.4. Soil Microbial Function

**Figure 4:**
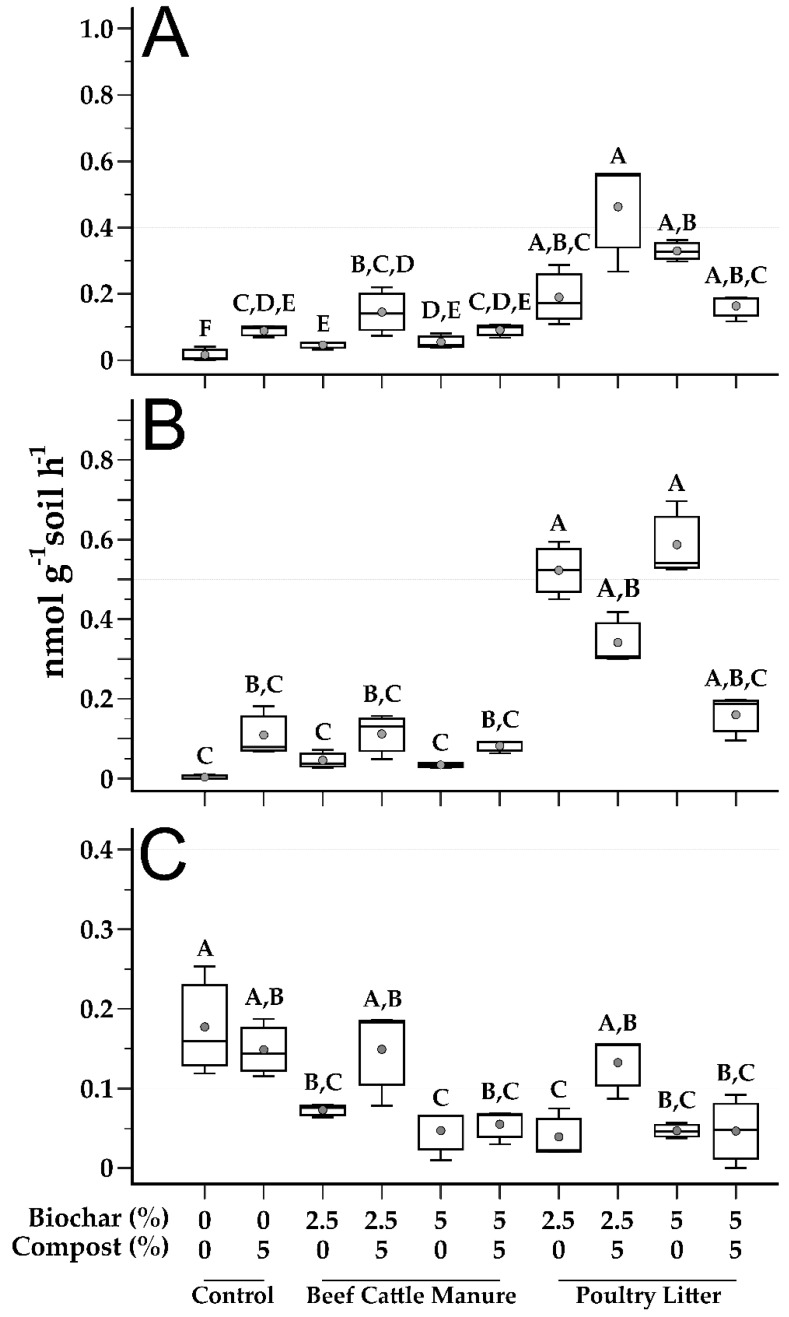
Soil extracellular enzyme activity. Top panel (**A**) is β-glucosidase, middle panel (**B**) is N-acetyl-β-d-glucosaminidase, and bottom panel (**C**) is acid phosphomonoesterase. Vertical axes for each enzyme are not shown on same scale. Gray circles are mean values, top and bottom of boxes are 75th and 25th percentile, respectively, median is center line, and whiskers are range of the data. Treatments that do not share a letter are significantly different (*p* < 0.05). Data are presented as untransformed, with statistical analysis based on log-transformed values.

**Figure 5:**
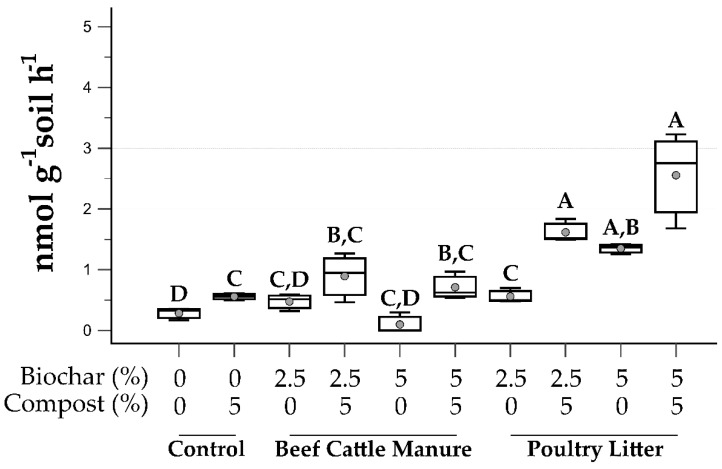
Soil intracellular enzyme activity as measured by esterase. Gray circles are mean values, top and bottom of boxes are 75th and 25th percentile, respectively, median is center line, and whiskers are range of the data. Treatments that do not share a letter are significantly different (*p* < 0.05). Data are presented as untransformed, with statistical analysis based on log-transformed values.

Microbial function was assessed, in the form of both extracellular (Figure 4) and intracellular (Figure 5) enzyme activity. Extracellular enzymes assayed were as follows: β-glucosidase (BG), used as an indicator of C cycling; N-acetyl-β-d-glucosaminidase (NAG), a chitinase involved in chitin degradation and used as an indicator of N cycling in soils; acid phosphomonoesterase (AcP), used as an indicator of P cycling. Esterase (EST), a hydrolase, is an intracellularly located enzyme; as one of the most abundant hydrolase enzymes found in soils, it was used to assess general microbial activity [48].

For BG, which is frequently implemented in soil health indices as an indicator of nutrient cycling, the lowest activity rate was exhibited in the control soil (mean: 0.02 nmol g^−1^ soil h^−1^; Figure 4A). All treatments exhibited significantly greater activity rates as compared to the unamended control. Maximal rates of BG activity were achieved with the 2.5% PL biochar with compost (mean: 1.45 nmol g^−1^ soil h^−1^; Figure 4A), which was significantly greater than the BG activity rates of all control and BC biochar treatments but did not significantly differ from the activity rates of the other three PL biochar treatments (Figure 4A). When comparing biochar treatments, with one exception (5% biochar with compost), BG activities were significantly greater in PL biochar treatments as compared to their BC counterparts. This result is similar to the earlier report by Ducey et al. [19] where PL treatments resulted in greater BG activity as compared to other treatments..

For NAG (Figure 4B), three PL treatments exhibited significantly greater enzymatic activity rates as compared to the unamended control—PL at 2.5% and 5% without compost, and PL 2.5% with compost (Figure 4B). No BC biochar treatments, with or without compost, differed significantly from the unamended control. Interestingly, for PL treatments, NAG activity significantly decreased in both instances where compost was added. This suppression of NAG activity upon addition of compost is potentially due to readily accessible organic N supplied by the compost [49].

The other extracellular enzyme, AcP (Figure 4C), showed an inverse pattern compared to the other two. For AcP, the unamended control soil showed the greatest rate of enzymatic activity (mean: 1.78 nmol g^−1^ soil h^−1^; Figure 4C). When biochar was amended into the mining impacted soils, of eight total treatments, six exhibited significantly lower enzymatic activity as compared to the unamended control. The two treatments with similar AcP activity rates to the unamended control were the 2.5% amendment rate BC and PL biochars with compost addition. This response is interesting because PL biochar has larger available P pools than BC biochar (Supplementary Table S1), and previous studies have demonstrated that P addition can lead to suppression of phosphatase activity [50]. Correlation between soil P concentrations and AcP activity showed an inverse relationship (*r* = −0.381, *p* = 0.03) supporting this observation.

To measure the impact of biochar and compost treatment on the overall microbial activity of mining impacted soils, the intracellular enzyme esterase, was assayed (Figure 5). With the exception of the two BC biochar treatments without compost, all treatments resulted in significantly greater activity as compared to the unamended control (mean: 0.28 nmol g^−1^ soil h^−1^; Figure 5). For both BC and PL biochars, amendment rates without compost resulted in lower activity rates as compared to similar treatments with compost, though this difference was only significant between the 2.5% PL biochar treatments with (mean: 0.90 nmol g^−1^ soil h^−1^) and without compost (mean: 0.56 nmol g^−1^ soil h^−1^) respectively (Figure 5). When comparing the two biochar feedstocks, with one exception (2.5% biochar without compost), esterase activity was significantly greater in the PL biochar treatments as compared to their BC counterparts. These results are similar to those observed in Ducey et al. [19], where mining impacted soils amended with beef cattle manure biochar did not show increased esterase activity, as compared to soils amended with poultry litter biochar. Maximal esterase activity was achieved in the 5% PL biochar with compost treatment (2.55 ± 0.79 nmol g^−1^ soil h^−1^) that resulted in a 9-fold increase in esterase activity, as compared to the unamended control soil. However, as previously mentioned in Ducey et al. [19], the esterase activity levels in these mining impacted soils are orders of magnitude lower than activities reported in healthier soils [51].

It should be noted that significant increases in esterase activity were achieved in these mining impacted soils after thirty-five days of treatment with PL biochar, compost, and maize-mediated phytostabilization. Given its biological nature, phytostabilization is a time intensive process. However, over the course of a month’s time, Sigua et al. [20] were able to document biochar-enhanced accumulation of Cd and Zn in maize tissue, and concomitant decreases of heavy metal concentrations in soil. Therefore, we hypothesize that soil microbial activities should continue to improve the longer mining impacted soils undergo phytostabilization management. The overall degree of increase however is a question that needs consideration. Are the current low activity rates a consequence of poor soil conditions (e.g., texture, low nutrient availability, high concentrations of heavy metals) that can be reversed with time and treatment, or are they a result of a microbial community incapable of activity rates comparable to neighboring soils that have never been impacted by mining activities?

This study represents the second report of the microbial response to amendment with manure-derived biochar and compost, in the mine impacted, remediated soils of the Oronogo-Duenweg Mining Belt. The first study in Ducey et al. [19], focused on the impact to microbial structure and function in these soils under growth with either switchgrass or buffalograss. The second study, reported here, used a similar (though smaller in scope) experimental design, to examine microbial responses under growth with maize. Patterns in microbial biomass were similar under all three plant types, with biomass increasing with addition of compost, and higher biomass values observed from soils amended with poultry litter-derived biochar as opposed to beef cattle manure feedstock. Differences were observed in the response of microbial community structure to plant type. Under maize, distinct community clusters were observed, separated by compost addition, biochar type, and rate. In comparison, under switchgrass and buffalograss [19], while microbial community structure continued to be influenced by biochar type and compost addition, it was not influenced by biochar amendment rate. Regarding microbial function, both studies reported similar observations for BG, NAG, and EST, with significantly increased enzyme activity rates often observed in soils amended with poultry litter derived biochar. While activity rates, in general, were lower than typically observed for healthy soils, biochar amendment did tend to improve activity rates when compared to unamended soil. Overall, these results indicate support of the hypothesis that microbial populations respond favorably to manure-derived biochars-particularly poultry litter-derived biochar—coupled with compost, under growth with maize.

These results also show potential when considering application at field-scale. As mentioned earlier, in the course of remediating these sites for Pb contamination, all Pb-laden soil was removed from the site. For the soils of the Oronogo–Duenweg Mining Belt, the Pb contamination was so pervasive that this resulted in loss of O, A, and B horizons. Microbial community structure is spatially sensitive, with significant changes occurring in microbial structure as a function of depth [52,53,54]. It has also been demonstrated that microbial activity is highest at the surface. For example, a study by Fang and Moncrieff demonstrated that microbial respiration was greatest in the top 8 cm [55]. It should be noted however that while the majority of microbial biomass is found at the soil surface, there remains a considerable microbial pool found between 25 cm and 2 m [56]. In systems where significant portions of topsoil are removed during remediation, a constraint is placed on biochar and compost for the selection and enhancement of microbial populations responsible for nutrient cycling. This selection is restricted to a limited, pre-existing pool of organisms that potentially display similar or greater activity rates when adjusted for biomass, and can be found in substantial numbers [57]. Therefore, depending upon the characteristics of individual sites, such as those represented in the soils of this study, it may be possible to remediate and restore landscapes without additional microbial inputs. Furthermore, it should be noted that considerable movement of microbes across landscapes [58], such as from nearby non-mining impacted (i.e., healthy) environments, can lead to introduction of new microbial species that may find the newly reconditioned soils favorable for colonization [59]. Additionally, if time is a constraining factor, the use of microbial inoculants [60] or locally-sourced microbial inoculum [61] can provide a boost to the restoration process.

## 4. Conclusions

In the Oronogo–Duenweg Mining Belt, for the successful restoration of mine impacted soils, additional treatment—post-remediation—will be required. These remediated soils, despite Pb removal, continue to display high concentrations of Cd and Zn, often at phytotoxic levels. The presence of these heavy metals, coupled with the loss of the O, A, and B horizons due to remediation, frustrate landscape revegetation, hamper phytostabilization efforts, and dissuade landowners from voluntarily participating in necessary remediation efforts. Treatment with biochar and compost have emerged as promising agents to address these issues. Previous studies using these mine impacted soils examined the influence of biochar and compost treatments on physicochemical characteristics for the successful sequestration of Cd and Zn to support plant growth. A companion study to these reports assessed the impact of these same treatments on biological characteristics of those same mine impacted soils. This study represents the culmination of those greenhouse studies, designed to address the question of whether these treatments impact soil physical, chemical, and biological characteristics in ways that benefit restoration efforts.

In this study, microbial biomass, structure, and enzyme activity all responded favorably to biochar and compost amendment under growth with maize, results similar to those demonstrated under different plant species. Overall, poultry litter biochar provided more beneficial impacts for mining impacted soils undergoing phytostabilization with maize. Poultry litter biochar led to lower available soil Cd and Zn concentrations, provided a larger boost to microbial biomass and soil enzymatic activity, and had a larger impact on microbial community structure. While the study was of a short duration, our results indicate that manure-based biochars can potentially provide long-term benefits and should be considered a reliable tool for future soil remediation and/or restoration efforts in mining impacted soils. Future research will now be directed at the application of these manure-derived biochars—specifically poultry litter-derived biochar—and compost amendments at field-scale levels to support restoration efforts utilizing plants native to southwestern Missouri.

## Figures and Tables

**Table 1:** Soil pH and EC means and standard errors.

Biochar ^1^	Biochar Rate (%)	Compost Rate (%)	pH ^2^	EC ^2,3^ (mS/m)
None	0	0	4.4 ± 0.1 ^g^	269.7 ± 55.2 ^e^
None	0	5	5.1 ± 0.0 ^e,f^	397.9 ± 85.9 ^d^
BC	2.5	0	5.1 ± 0.1^f^	262.2 ± 39.9 ^e^
BC	2.5	5	5.3 ± 0.1 ^d,e^	473.9 ± 35.1 ^c,d^
BC	5	0	5.3 ± 0.2 ^d^	437.1 ± 24.6 ^c,d^
BC	5	5	5.9 ± 0.1 ^c^	559.6 ± 72.4 ^c^
PL	2.5	0	5.5 ± 0.2 ^d^	1425.7 ± 281.7 ^b^
PL	2.5	5	5.9 ± 0.0 ^c^	1577.0 ± 419.4 ^b^
PL	5	0	6.3 ± 0.0 ^b^	2767.0 ± 600.5 ^a^
PL	5	5	6.6 ± 0.0 ^a^	2765.7 ± 322.5 ^a^

^1^ BC = beef cattle manure; PL = poultry litter. ^2^ Treatments that do not share a letter are significantly different (*p* < 0.05). ^3^ Data are presented as untransformed, with statistical analysis based on log-transformed values.

## Data Availability

Raw data supporting the reported results has been uploaded to the Science Data Bank under DOI 10.11922/sciencedb.01185.

## Supplementary Materials

The following are available online at https://www.mdpi.com/article/10.3390/microorganisms9122545/s1, Supplementary Table S1: Soil characterization data for secondary matrix for NMS analysis.
