# Supplementary material for: Microbial Response to Phytostabilization in Mining Impacted Soils Using Maize in Conjunction with Biochar and Compost
Source: Microorganisms. 2021 Dec 9;9(12):2545. doi: 10.3390/microorganisms9122545 (PMC8707346; doi:10.3390/microorganisms9122545)
Supplement: Supplementary file 1 [file microorganisms-09-02545-s001.zip › microorganisms-1422633-supplementary.pdf]

Supplementary Table S1. Soil characterization data for secondary matrix for NMS analysis

Biochar Type	Biochar Rate (%)	Compost Rate (%)	Cd (mg/kg)	Cu (mg/kg)	K (mg/kg)	Mn (mg/kg)	Mg (mg/kg)	Na (mg/kg)	P (mg/mg)	Zn (mg/kg)	pH	EC
None	0	0	1.93	0.21	8.57	1.52	0.00	7.87	0.28	58.39	4.41	222.2
None	0	0	1.94	0.17	8.69	1.52	0.00	7.51	0.15	58.55	4.46	256.6
None	0	0	2.31	0.23	9.42	1.84	0.00	8.35	0.12	69.23	4.34	330.2
None	0	5	1.62	0.20	27.09	4.39	6.57	14.75	0.27	61.55	5.13	341.7
None	0	5	1.71	0.22	25.11	4.14	3.63	15.73	0.58	63.79	5.07	496.7
None	0	5	1.20	0.29	20.44	3.03	0.00	12.14	0.68	46.01	5.10	355.2
BC	2.5	0	1.70	0.15	14.57	2.38	0.00	11.59	0.09	54.51	4.95	268.7
BC	2.5	0	1.63	0.16	15.39	2.21	0.00	9.65	0.21	52.41	5.22	299.1
BC	2.5	0	1.92	0.17	17.06	2.60	0.00	11.80	0.12	62.03	5.05	220.0
BC	2.5	5	1.16	0.33	29.23	4.15	11.17	17.27	0.67	44.84	5.41	511.2
BC	2.5	5	1.07	0.25	24.73	3.50	4.74	15.17	0.24	39.69	5.25	468.8
BC	2.5	5	1.08	0.29	26.20	3.50	4.83	17.56	0.53	40.77	5.17	441.6
BC	5	0	1.69	0.15	23.55	3.52	0.00	13.71	0.00	53.34	5.20	434.2
BC	5	0	1.82	0.17	23.56	3.78	0.00	14.62	0.00	57.84	5.17	463.0
BC	5	0	1.54	0.21	21.50	3.33	0.00	13.33	0.12	50.26	5.57	414.0
BC	5	5	0.69	0.24	32.24	3.63	16.87	16.98	0.57	25.06	5.76	476.0
BC	5	5	0.93	0.21	40.52	4.70	28.98	19.37	0.39	32.76	5.98	599.7
BC	5	5	1.21	0.25	40.53	4.99	29.92	20.77	0.82	40.74	6.01	603.0
PL	2.5	0	4.10	0.79	142.90	7.50	85.70	307.92	1.23	112.09	5.50	1.736
PL	2.5	0	3.69	0.85	145.68	6.81	77.38	279.04	1.48	101.33	5.28	1.355
PL	2.5	0	2.37	0.63	115.57	4.69	49.47	222.80	1.30	68.64	5.60	1.186
PL	2.5	5	1.55	0.85	162.08	7.02	102.06	245.28	2.13	48.33	5.83	2.043
PL	2.5	5	1.59	0.77	148.45	6.90	93.03	239.34	2.02	50.49	5.86	1.458
PL	2.5	5	1.35	0.64	131.65	5.84	76.27	222.71	1.60	43.79	5.86	1.230
PL	5	0	1.18	1.25	402.85	4.57	150.79	457.62	3.06	20.41	6.32	3.171
PL	5	0	1.19	1.35	385.36	5.02	161.47	464.09	3.46	21.82	6.31	2.077
PL	5	0	1.22	1.38	448.04	4.87	173.50	497.22	3.80	19.49	6.37	3.053
PL	5	5	0.66	1.30	523.24	3.86	173.88	421.28	4.01	11.31	6.61	2.766
PL	5	5	0.78	1.68	646.16	4.20	228.90	543.54	5.41	12.26	6.61	3.088
PL	5	5	0.45	1.18	401.64	2.98	126.20	308.93	7.42	8.50	6.62	2.443
